# Youth Dating Violence, Behavioral Sensitivity, and Emotional Intelligence: A Mediation Analysis

**DOI:** 10.3390/healthcare11172445

**Published:** 2023-08-31

**Authors:** María Pilar Salguero-Alcañiz, Ana Merchán-Clavellino, Jose Ramón Alameda-Bailén

**Affiliations:** 1 Basic Psychology Area, Department of Clinical and Experimental Psychology, University of Huelva, 21007 Huelva, Spain; pilar.salguero@dpsi.uhu.es; 2 Social Psychology Area, Department of Psychology, University of Cádiz, 11519 Cádiz, Spain; 3 INDESS (Research University Institute for Sustainable Social Development), University of Cádiz, 11406 Jerez de la Frontera, Spain

**Keywords:** behavioral sensitivity, emotional intelligence, violence in young couples

## Abstract

Intimate partner violence is a multidimensional phenomenon encompassing psychological, physical, and sexual components. Violence in young couples is common in our society. This kind of violence is usually bidirectional, which adds to its complexity. This study aimed to explore how victimization (in three dimensions: non-abuse, technical mistreatment, and mistreatment) and perpetration (in two dimensions: non-perpetrator and perpetrator) are related to the BIS (Behavioral Inhibition System)/BAS (Behavioral Approach System), and it also evaluated if the dimensions of emotional intelligence (EI) (emotional attention, clarity, and regulation) mediate this relationship. Violence was evaluated in 272 young volunteer participants, as well as BIS/BAS behavioral sensitivity and perceived emotional intelligence. The correlations between these variables were analyzed, and a mediation analysis was also conducted. The results show that victimization (of the sexual and coercive type) was associated with less BAS activation, while victimization (of the sexual, humiliation, and detachment types) was associated with less BIS activity. All types of victimization were associated with less EI, specifically with less emotional clarity. Aggression (of the sexual, humiliation, detachment, and coercion types) was related to lower BAS and higher BIS sensitivity. Detachment aggression was associated with low emotional clarity. In conclusion, relationships between victimization and perpetration are evidenced in terms of BIS/BAS sensitivity and EI. Specifically, the dimension of EI emotional clarity acts as a mediator of BIS activation in victims of detachment.

## 1. Introduction

Intimate partner violence is a multidimensional phenomenon that encompasses psychological, physical, and sexual components [1], and it can emerge at any time in the relationship. Intimate partner violence is increasingly appearing at younger ages [2] and in both sexes [3]. 

Violence within couples is a complex issue with various elements that should be considered comprehensively. This phenomenon extends beyond traditional stereotypes of male-perpetrated physical violence, where men are the aggressors and women the victims [4,5]. This approach is reductionist and is not appropriate for addressing a phenomenon as complex and heterogeneous as couple violence. In this paper, we focus on some factors (behavioral sensitivity and emotional intelligence) that may be important but are not the only ones that potentially contribute to dating violence.

Violence in young couples is different from that in adult couples [4]. Recent studies on violence in young couples indicate that both men and women are equally likely to perpetrate violence [6,7,8,9,10,11,12]. Thus, both sexes have the same predisposition to perpetrate violence [10]. Recently, Conroy et al. [13] demonstrated that problematic attitudes towards violence are not limited to men but also exist in women. Furthermore, bidirectional violence, where both partners act as both aggressors and victims interchangeably, is currently the most prevalent form [5,6,7,8,11,13,14,15,16,17,18,19]. This is more pronounced in psychological attacks compared to physical ones [14]. Nevertheless, there are sex differences, as women perpetrate both psychological and physical violence, while physical violence prevails among men [9,10,11,20,21,22]. This signals a need to raise awareness of abuse perpetrated by women against men so that they can ask for help without feeling ashamed.

Violence is not a spontaneous or natural phenomenon [5]. Men and women intentionally learn to use violent behaviors to harm their respective partners [23,24]. Therefore, learning plays a crucial role in the emergence and persistence of violent behavior. Violent behavior also has emotional and motivational components, akin to other learned behavior. Additionality, it is essential to not only examine the cognitive and emotional variables associated with the aggressor’s profile, as is traditionally done, but also, according to more recent theoretical perspectives, the determinants of the victim’s profile [25].

In this context, the theory of behavioral sensitivity [26,27,28] provides an integrated approach for understanding behavior from a personality perspective, encompassing emotion, motivation, and learning. This theory is based on two complementary behavioral systems: the Behavioral Activation System (BAS) and the Behavioral Inhibition System (BIS). Each system involves different neural correlates specialized in detecting, processing, and responding to certain stimuli [29]. These motivational systems can trigger emotional and behavioral responses in threatening situations [30,31].

The BAS specializes in processing information related to incentives and rewards, leading to positive feelings like hope and euphoria and motivating approach behaviors. Individuals with high BAS sensitivity exhibit this response even to small incentives [32]. Conversely, the BIS system processes information related to punishment, aversive stimuli, and threats, causing arousal and heightened attention to threats when danger signals are present. People with high BIS sensitivity experience distress and anxiety even in response to minimal threats [32].

The relationship between BIS/BAS behavioral sensitivity and intimate partner violence has not been systematically explored. However, Meyer et al. [31] examined the relationship between the BIS/BAS scales and the hypothetical threat of losing a partner, finding significant associations between the activations of both systems (BIS and BAS) and the threat of partner loss. These findings are significant as they reveal a close connection between the threat of partner loss and the different psychological profiles of both victims of abuse and perpetrators of different types of violence.

Hence, the behavioral sensitivity theory [29] highlights the fundamental role that motivations and emotions play in learning. The construct of emotional intelligence (EI) is also relevant in this context as it reflects the inseparable link between cognition and emotion [33]. The most widely accepted definition of EI defines it as “the ability to accurately perceive, assess and express emotions, the ability to access and/or generate feelings that facilitate thought; the ability to understand emotions and emotional knowledge and the ability to regulate emotions promoting emotional and intellectual growth” ([34], p. 5).

The association between EI and violence in young couples has been previously described. García González and Quezada [35] found that EI enhances satisfaction in couple relationships by aiding in the resolution of inherent conflicts, while a low level of EI is associated with stress and violence in relationships. This link between EI and violence has also been emphasized by Zapata [36], showing a significant negative correlation between EI and the dimensions of coercion, physical, detachment, and humiliation perpetration. This aligns with the proposal of Moreno et al. [37], which consists of the implementation of programs based on the acquisition of EI skills to reduce and/or prevent violence in young couples.

Furthermore, various studies have described the association between EI and affective states, indicating that high EI is associated with a positive mood, while low EI is linked to a negative mood [37,38,39,40].

The BIS/BAS systems are also related to EI, with high EI being characterized by reward sensitivity (BAS) and low EI being associated with low BIS activation [38]. This relationship between the BIS/BAS systems and EI appears to involve mediation, wherein EI modulates the effects of the BIS/BAS systems on emotions, feelings, and moods.

Hence, it is plausible that both the BIS/BAS systems and EI are variables that play a role in the behavior of couples, both victims and aggressors, who engage in violence.

Therefore, this work aimed to analyze the relationship between the BIS/BAS systems and EI in the violent behavior of young couples, as well as the directionality of this relationship, to understand the risk and vulnerability factors associated with couple violence, both in both victims and aggressors. This understanding can lead to more targeted and effective preventive interventions.

Consequently, the hypothesis of this study posits that both victims and aggressors will score higher on the BIS and lower on the BAS. Additionally, both victims and aggressors are expected to exhibit low levels of EI.

## 2. Materials and Methods

### 2.1. Participants

The sample included 272 Spanish volunteer participants, with a mean age of 20.97 years (*SD* = 2.52), ranging from 19 to 30 years old (82.7% women). Approximately half of the participants had studied at the university level (52.2%), 1.8% of them had a master’s degree, 33.1% had a bachelor’s degree, 12.5% had vocational training, and 0.4% had completed secondary education.

### 2.2. Procedure

Data were collected using self-administered online questionnaires, using the random sampling method. Participation was anonymous and the data were recorded confidentially. All participants were informed of the study objectives and the possibility of dropping out of the study at any time. The study was conducted according to the 1975 Declaration of Helsinki of the World Medical Association (amended by the 64th General Assembly, Fortaleza, Brazil, October 2013), and all participants signed the written informed consent form.

### 2.3. Instruments

An ad hoc questionnaire was developed to record the sociodemographic information of the participants. The requested data were sex, age, and current educational level.

Revised Dating Violence Questionnaire (DVQ-R) [40]: This questionnaire evaluates two categories in the evaluation of violence: victimization and perpetration. Scores are obtained on five dimensions for each category: alienation, humiliation, coercion, physical violence, and sexual violence. DVQ-R includes 20 items on a Likert-type scale with five response options, ranging from 0 (never) to 4 (all the time). The internal consistency of this questionnaire for the five scales ranges between 0.64 and 0.74 (Cronbach’s alpha, α), and for the total scale, the consistency is α = 0.85. The internal consistency found in our sample for the five scales ranged between α = 0.5 and 0.7 [40,41,42]. In addition, the perception of abuse was analyzed through three yes/no questions: Are you or have you been afraid of your partner? Do you feel or have you felt trapped in your relationship? Have you ever felt mistreated in your relationship? An α value of 0.6 was obtained for the three items.

The Sensitivity to Punishment (SP) and Sensitivity to Reward (SR) Questionnaire (SPSRQ; [43]): This is a Spanish version of the measurement of the BIS/BAS systems. SPSRQ consists of 48 dichotomous items (yes–no), and it is divided into two 24-item scales: Sensitivity to Punishment (SP) as a measure of the BIS and Sensitivity to Reward (SR) as a measure of the BAS. The reliability of the scale is adequate, with the SP scale showing an α value of 0.83 and the SR scale showing an α value of 0.76 [44]. In our sample, there was an alpha value of 0.8 for SP and 0.7 for SR.

Trait Meta-Mood Scale, TMMS-24 [45]: This scale includes 24 Likert-type items, ranging from 1 to 5. It is divided into three dimensions of perceived emotional intelligence, each with 8 components: emotional attention (ability to identify one’s own emotions and the emotions of others and ability to know how to express emotions), emotional clarity (understanding of emotions), and emotional repair or regulation (ability to manage emotions). The reliability and validity indices reported are adequate [46], and these indices were also adequate in our sample. Reliability in attention was α = 0.8, α = 0.9 in clarity, and α = 0.8 in regulation.

### 2.4. Statistical Analysis

In the preliminary analyses, descriptive statistics (percentages, means, and standard deviations) were calculated, and mean difference *t*-tests (for the variables with two response alternatives) and analysis of variance (ANOVA), for variables with two or more response options (violence group), were conducted to analyze significant differences. Post hoc tests were performed for respective comparisons. Cohen’s d was calculated for standardized mean differences, and based on the values obtained, an effect size of less than 0.2 was considered “small”, between 0.5 and 0.8, the effect size was considered “medium”, and for any value upwards of 0.8, the effect size was considered “large” [47]. Pearson correlations were calculated between the study variables. The internal consistency of the scales was analyzed using Cronbach’s alpha coefficient.

**Figure 1:**
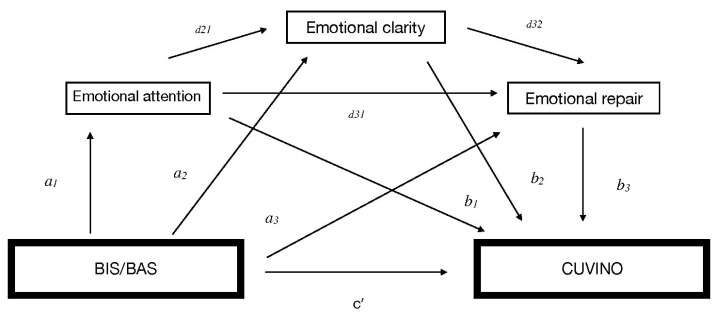
Conceptual and statistical scheme of the mediation of EI in the relationship between the BIS/BAS and the CUVINO factors for victimization and aggression.

The SPSS 25.0 statistical package was used, and according to the macro Process [48], the mediation analysis was established with a 95% confidence interval and a number of bootstrapping samples of 10,000. The estimates of each analysis were calculated through their respective unstandardized regression coefficients (coeff), their standard errors (SEs), t-values and their significance levels (p), and the different values of the lower limit (LLCI) and upper limit (ULCI) of the confidence interval. The interpretation of significance was performed through the values of each LLCI and ULCI. Therefore, when the number 0 was found between this interval, it confirmed that this particular result was not significant. The serial mediation analysis was conducted using model 6 and analyzed whether the effect of the independent variable (X) (BIS) on the dependent variable (Y) (CUVINO categories: victim/aggressor) may be mediated by the mediating variables (M1; M2; M3), that is, the perceived emotional intelligence, with its three dimensions (attention, clarity, and emotional repair), including as covariates the sex and age variables. As shown in Figure 1, parameter (*c*′) indicates the direct effect of X on Y, controlling for the mediating variable, (*a*) indicates the direct effect of X on M, (*b*) is the direct effect of M on Y, the indirect effect (*ab*) is the effect through the mediating variable, and the total effect (*c*) is the sum of the direct and indirect effects, when the mediator is excluded from the regression analysis.

## 3. Results

### 3.1. Descriptive Analyzes

Table 1 shows the count and percentage for each group according to the dimensions of the CUVINO questionnaire. A total of 83.1% of the sample reported some type of violence. Most of the violence reported was bidirectional (70.6%); that is, victim and aggressor were indistinctly reported by men and women. One-way violence was considerably lower (aggressor and non-victim: 3.7%; victim and non-aggressor: 12.5%). No victim or aggressor was reported in 13% of the total sample. 

Regarding the distribution by sex, there were no statistically significant differences for the victim profiles *(X*^2^ = 1.407; *p* = 0.495), and no relationship was observed for aggression profiles either (*X*^2^ = 0.591, *p* = 0.442).

Table 2 shows the descriptive statistics corresponding to age, emotional intelligence, and BIS/BAS for each group according to the type of intimate partner violence and total sample.

In the victimization profile (abuse, technical abuse, and no abuse), significant differences regarding age were observed (F = 6.182; *p* = 0.002). Specifically, the maltreated were older than the non-maltreated (*p* = 0.045, *d* = 1.1) and the technically mistreated (*p* = 0.002, *d* = 1.2). Regarding behavioral sensitivity, significant differences were found between groups (abused, technical abuse, and no abuse) for the BAS variable (F = 3.170; *p* = 0.044), but no differences were observed in the BIS (F = 1.069; *p* = 0.345). Finally, regarding the EI dimensions, differences were observed between the victimization groups (mistreated, technical abuse, and non-abuse) in two dimensions: emotional clarity (F = 4.746; *p* = 0.009) and emotional repair (F = 3.426; *p* = 0.034). Pairwise comparisons showed differences between the abused and non-abused groups in the following dimensions: emotional clarity (*p* = 0.014; *d* = 3.71) and emotional repair (*p* = 0.042; *d* = 3.1), and between technical abuse and abused in the emotional clarity dimension (*p* = 0.046, *d* = 2.3). In all cases, the maltreated group obtained worse scores on the scales analyzed, followed by the technical maltreatment group, and the best results were observed in the non-abuse group. In summary, no differences between abused and non-abused were observed in the BIS. Abused people’s scores were lower on the BAS scale, and they also obtained lower scores on the EI dimensions of clarity and repair. The scores of the technically mistreated group were lower than those of the mistreated group.

In the aggression profile, significant differences were observed regarding the factor age, with the aggressors being older than the non-aggressors (t = −2.965; *p* = 0.003). On the other hand, in terms of behavioral sensitivity, significant differences were observed between perpetrators and non-perpetrators on the BAS scale (t = 3.416; *p* = 0.001; *d* = 1.85), with non-aggressors showing higher scores. On the BIS scale, no significant differences were observed (t = 0.299; *p* = 0.765: *d* = 0.23). Finally, regarding the aggressors and non-aggressors EI, no significant differences were observed in the attention and emotional repair dimensions, although there was a trend of higher scores in the non-abused group compared with the abused group (t = 1.914; *p* = 0.057; *d* = 1.84).

The correlations between the three emotional intelligence dimensions (attention, clarity, and emotional repair), the BIS/BAS, age, and the two dimensions of the CUVINO (victimization and perpetration) were also analyzed (see Table 3). 

Regarding the victimization profile, a negative correlation was observed between the BAS and the sexual- and coercive-type victims. The BIS negatively correlated with being a sexual victim, humiliation, and detachment. Regarding the EI dimensions, there was a negative correlation between the clarity dimension and each type of victim, while the emotional repair dimension was negatively correlated with being a sexual victim and a victim of detachment. Therefore, the lower the BIS score, the higher the victimization, and the higher the victimization, the lower the EI. That is, in the couple violence victims, the BIS and EI variables correlated negatively.

In the perpetration profile, the BAS was negatively correlated with being a sexual aggressor, humiliation, detachment, and coercive violence. However, the BIS was not correlated with the types of aggressors. Regarding the EI dimensions, a negative correlation was observed between attention and emotional clarity in detachment aggression. That is, the higher the detachment aggression, the lower the attention and emotional clarity. In the emotional repair dimension, no correlation was observed with any type of aggression.

### 3.2. Mediation Analysis

After the preliminary and correlation analyses, we aimed to study the mediation process of EI in the relationship between the BIS/BAS and the role of the victim or aggressor. In the mediation analysis, the mediator variable (EI) should correlate both with the dependent variable (victimization/perpetration) and the independent variable (BAS/BAS).

According to our analyses, EI was not correlated with the BAS, so this variable was not included in the mediation analyses. However, EI was significantly correlated with the BIS and victimization, but the BIS was not correlated with perpetration. Therefore, the mediation analysis was conducted to explore if the EI mediates the effects of the BIS on the related types of victims, that is, sexual, humiliation, and detachment.

Thus, three serial mediation models were analyzed to determine if the BIS scores and EI dimensions are related to sexual victimization (model 1), humiliation victimization (model 2), and detachment (model 3). Age and sex were considered covariates (Table 4). 

We detail here the results of the different linear regression analyses considering the EI dimensions and the independent variable (BIS). Regarding emotional attention, the percentage of variance explained by the BIS (*a*_1_) and the covariates was 4.06%, although only the factor sex was significant (*B* = 2.414; t = 2.438; *p* = 0.0154). Regarding emotional clarity, the percentage of variance explained by the BIS and the covariates was 31.54%. In this case, significant differences were observed in the BIS (*a*_2_) and emotional attention (*d*_21_), with the factors sex and age showing no significance (*p* > 0.05). Concerning emotional repair, the percentage of variance explained by the BIS was 27.90%, with the covariate sex being significant (*B* = −2.147; t = −2.192; *p* = 0.029), as well as the variables BIS (*a*_3_), attention emotional (*d*_31_), and emotional clarity (*d*_32_) (Table 3).

Regarding the results of the multiple linear regression analyses, considering EI and the BIS as predictor variables, the three types of victims with significant correlations were included. 

First, for model 1 (sexual victimization), the BIS (*B* = −0.0242; t = −1.979; *p* = 0.0488) and the covariate age (*B* = 0.0558; t = 2.283; *p* = 0.0232) were significant factors, and they explained 6.26% of the total variance. In this model, the total effect of the BIS was significant. In addition, in this model, direct, but not indirect, effects of the BIS were observed.

Second, for model 2 (victimization by humiliation), the total variance explained was 3.98%, although here there were no significant differences, and we only found a trend of significance in emotional clarity (*b*_2_) (F = 0.0936; *p* = 0.093). In this model, the total effect of the BIS was significant, while neither direct nor indirect effects were observed (Table 3).

**Figure 2:**
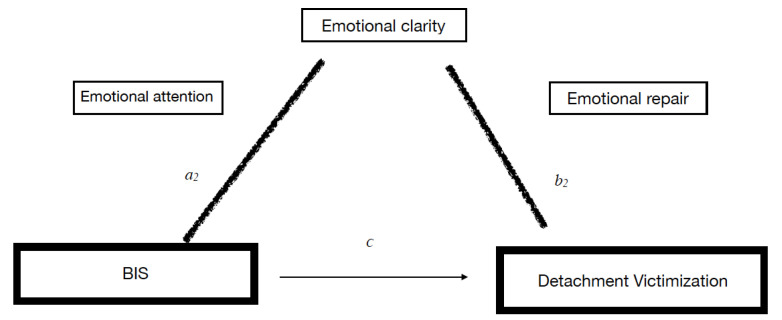
Mediation of EI in the relationship between the BIS/BAS and the CUVINO factors for victimization and aggression. Indirect effects corresponding to Model 3: BIS on detachment victimization.

Finally, for model 3 (detachment victimization), a significant effect was observed in the multiple linear regression analysis (*F* = 4.434; *p* = 0.0003). Emotional clarity (*b*_2_) resulted in a significant variable explaining 9.12% of the total variance of the model in this type of victim (Table 3). In this model, the total effect of the BIS was significant, but no direct effects were observed. Lastly, in this model, we observed indirect effects of the BIS (X) on detachment victimization (Y), and these effects were mediated by emotional clarity (Figure 2 and Table 3). Therefore, the emotional clarity dimension in the victims due to detachment can be considered a mediator of the BIS. Thus, low levels of BIS are associated with poor emotional clarity and more indicators of victimization due to detachment.

## 4. Discussion 

According to the descriptive analyses, the majority of reported abuse cases appear to be bidirectional, a trend consistent with previous studies [5,6,7,8,10,11,14,15,16,17,18,19]. This reciprocal pattern of violence is associated with the absence of discernible differences between men and women, both in terms of victim and aggressor profiles. In other words, both men and women can interchangeably be victims or aggressors in intimate partner violence.

Regarding the victimization profiles, distinctions emerged among different types of victims concerning behavioral sensitivity. Victims exhibited lower scores on the BAS scale, indicating a reduced sensitivity to incentives and goal-directed behavior. Consequently, victims reported lower levels of happiness and positive moods [32]. This is also true concerning the aggression profile, but in the opposite direction; that is, higher scores were found in the BAS scores of people who do not mistreat their partners. These people are sensitive to rewards, feel hope and euphoria, aim at accomplishing goals and finding happiness in their achievements, and, therefore, show more affection, feelings, and positive emotions [32].

In terms of EI, victims who reported abuse and technical mistreatment exhibited lower emotional clarity and emotional repair abilities. This implies a deficiency in understanding their own emotions and the emotions of others, which may hinder their ability to regulate emotions effectively (self-regulation). Consequently, victims with low EI experience personal discomfort. It is evident that victims tend to possess lower EI, aligning with the conclusions of Moreno et al. [37], who emphasize the importance of EI skills in preventing violence in young couples. In contrast, individuals who do not engage in aggression, particularly those who refrain from physical violence against their partners, displayed higher emotional clarity. This means they have a better grasp of their own emotions and the emotions of others, consistent with the results of Zapata [36], who demonstrated significantly lower EI levels in individuals perpetrating violence against their partners.

Regarding the correlations in the victimization profile, a negative correlation was observed between the BAS scores for sexual and coercive victims. High levels of sexual and coercive victimization correlated with low BAS activation. This is consistent with the results of Meyer et al. [31] since they reported that the BAS is also affected by situations of interpersonal threat because these involve the loss of rewards and incentives (in this case, the couple’s relationship). Furthermore, sexual victimization, humiliation, and detachment correlated negatively with the BI, indicating that these types of victims do not perceive violence with their partners as a threat and do not pay attention to danger signals, even in high-threat situations [49]. It is worth noting that there is no established causal relationship between low BIS activation and high victimization. Low BIS levels may be linked to EI since all victim types exhibited a negative correlation with emotional clarity, implying a reduced ability to understand their own emotions and those of others. This lack of emotional clarity might explain why victims do not perceive intimate partner violence situations as threatening and do not process danger signals effectively. Concerning emotional repair, which involves self-regulation and the management of personal emotions, the same trend is observed. Victims of sexual victimization exhibited a negative correlation between detachment and the BIS. It is possible that low BIS levels in sexual victims are related to low EI levels because threatening situations may not be processed as such.

On the other hand, within the aggression profile and terms of behavioral sensitivity, sexual, humiliation, detachment, and coercive aggressors show lower scores in the BAS, which means that they do not effectively process information related to incentives and rewards, and as a result, they do not generate positive feelings such as hope and euphoria. In this aggression profile, we also found higher BIS scores; that is, these aggressors process the information as highly threatening and triggering anxiety and excitement, and they focus attention on danger signals [32]. The EI of the aggressors due to detachment negatively correlated with attention and emotional clarity. We found that low EI is associated with aggression, which means that the aggressors, at least due to detachment, have a poor ability to understand their own emotions and the emotions of others, and this low emotional clarity may be related to the use of violence as a way of resolving conflicts in the couple, in the absence of healthy strategies.

Finally, in the context of mediation analysis, our results demonstrate that in victims of detachment violence, EI, particularly the emotional clarity dimension, acts as a mediator of the BIS. Previous studies have shown that BIS activity moderates the relationship between threat and anxiety [49]. Meyer et al. [31] highlighted the effect of BIS sensitivity in threatened relationships, noting that low partner threat levels are not associated with distress, even with high BIS levels, whereas high threat levels and high BIS levels are closely linked to distress. High BIS sensitivity has previously been associated with distress in various stressful situations [49,50,51,52,53]. The perception of situations as stressful or highly threatening appears to be essential for triggering anxiety.

Our results suggest that threat perception might in turn be influenced by EI, specifically by emotional clarity. Victims of couple violence due to detachment exhibited low emotional clarity, implying a diminished ability to understand personal emotions and the emotions of others. This may prevent victims from interpreting violence as a threat to the relationship or as a stressful situation in general. Consequently, the BIS response is not triggered, as the situation is not perceived as dangerous to personal well-being [31].

This study had certain limitations. Expanding the sample to include specific sex and age groups and collecting data from nationally representative samples would be beneficial. Longitudinal studies would also be valuable, as cross-sectional studies may limit the generalizability of the findings. Additionally, it is important to acknowledge that self-reports and observational studies can introduce biases.

Nevertheless, this study significantly contributes to understanding the motivational and emotional processes involved in dating violence, shedding light on potential psychological interventions.

## 5. Conclusions

The findings of this study reveal that there are no significant differences in young couple violence between sex, as both men and women can be victims and aggressors interchangeably, demonstrating a bidirectional pattern of violence. However, differences are observed concerning age, as both victimization and perpetration rates increase with age.

Regarding behavioral sensitivity, both victims and aggressors show low BAS sensitivity, while individuals who do not engage in abuse behavior towards their partners display high BAS sensitivity.

In terms of EI, it has been observed that victims of abuse and technical abuse, as well as perpetrators, tend to have lower EI levels compared to individuals who do not commit violence against their partners, who show higher levels of EI.

Additionally, sexual and coercive victimization are associated with reduced BAS activation, whereas sexual, humiliation, and detachment victimization are linked to lower BIS activation. Furthermore, all types of victimization are correlated with less emotional clarity. In the case of aggressors, including those involved in sexual, humiliation, detachment, and coercion, lower BAS activation and higher BIS activation are observed. Detachment aggression is specifically associated with poor emotional clarity.

Finally, the mediation analyses have revealed that emotional clarity, an EI dimension, acts as a mediator of BIS activation in victims of detachment. Based on these results, the implementation of programs for the acquisition of EI skills in young people of both sexes might be a useful approach for this kind of victim. Specifically, we consider it important that nursery school programs train students in EI, as this is fundamental to being able to identify and understand their own emotions as well as those of the people around them. In addition, psychotherapists need to consider the important tool that EI represents in the fight against intimate partner violence, both for men and women.

## Figures and Tables

**Table 1:** Count and percentage for each group according to the dimensions of the CUVINO questionnaire.

	Victimization Profile			Aggression Profile	
	Non-Abuse	Technical Mistreatment	Mistreatment	Non-Perpretator	Perpetrator
Overall	46 (16.9%)	153 (26.8%)	73 (56.3%)	70 (25.7%)	202 (74.3%)
Men	6	30	11	10	37
Women	40	123	62	60	165

**Table 2:** Descriptive statistics corresponding to age, emotional intelligence, and the BIS/BAS for each group according to the type of couple violence and total sample.

	Victimization Profile						Aggression Profile				Overall	
	Non-Abuse		Technical Mistreatment		Mistreatment		Non-Perpretator		Perpetrator			
	M	*SD*	M	*SD*	M	*SD*	M	*SD*	M	*SD*	M	*SD*
Age	20.70	2.45	20.63	2.28	21.84	2.83	20.31	1.92	21.19	2.66	20.97	2.51
Emotional attention	30.54	6.11	29.99	6.03	29.73	6.31	30.86	5.85	29.72	6.18	30.01	6.11
Emotional clarity	28.04	6.83	26.71	6.79	24.32	7.13	27.67	6.35	25.83	7.14	26.30	6.98
Emotional repair	28.15	6.60	25.50	6.95	24.98	6.55	26.83	6.52	25.46	6.95	25.81	6.85
BIS	35.76	6.05	35.03	5.81	34.22	5.35	35.11	6.10	34.88	5.61	34.94	5.73
BAS	39.48	3.17	38.32	4.09	37.60	4.10	39.70	3.89	37.85	3.92	38.32	3.99

**Table 3:** Correlation between emotional intelligence, BIS/BAS, and CUVINO factors for victimization and aggression.

		1	2	3	4	5	6	7	8	9	10	11	12	13	14
1. Emotional attention		-													
2. Emotional clarity		0.443 **	-												
3. Emotional repair		0.200 **	0.436 **	-											
4. BIS		−0.134 *	0.277 **	0.371 **	-										
5. BAS		−0.073	0.045	0.044	0.031	-									
Victim	6. Physical	−0.049	−0.125 *	0.008	−0.024	−0.09	-								
	7. Sexual	0.002	−0.142 *	−0.140 *	−0.175 **	−0.128 *	0.492 **	-							
	8. Humiliation	−0.003	−0.147 *	−0.094	−0.142 *	−0.044	0.400 **	0.365 **	-						
	9. Detachment	−0.019	−0.247 **	−0.192 **	−0.186 **	−0.11	0.232 **	0.303 **	0.447 **	-					
	10. Coercion	−0.013	−0.139 *	−0.049	−0.082	−0.202 **	0.378 **	0.386 **	0.421 **	0.283 **	-				
Aggressor	11. Physical	−0.001	0.016	0.018	0.095	−0.09	0.440 **	0.255 **	0.198 **	0.173 **	0.168 **	-			
	12. Sexual	−0.021	−0.039	−0.012	0.037	−0.137 *	0.229 **	0.353 **	0.153 *	0.049	0.174 **	0.251 **	-		
	13. Humiliation	−0.013	−0.086	−0.076	0.005	−0.197 **	0.056	0.067	0.519 **	0.282 **	0.168 **	0.201 **	0.178 **	-	
	14. Detachment	−0.148 *	−0.214 **	−0.021	−0.041	−0.154 *	0.066	0.151 *	0.252 **	0.443 **	0.252 **	0.114	0.127 *	0.242 **	-
	15. Coercion	0.091	0.006	−0.117	−0.07	−0.197 **	0.104	0.136 *	0.233 **	0.233 **	0.500 **	0.312 **	0.098	0.274 **	0.185 **

* *p* < 0.05; ** *p* < 0.00.

**Table 4:** Results of the analysis of mediation of emotional intelligence in the relationship between BIS and sexual victimization (model 1), humiliation (model 2), and detachment (model 3), including as covariates age and sex.

Model 1 (Sexual Victimization)						
Path	Coefficient	HE	BootLLCI	BootULCI	t	*p*
Total effect (*c*)	−0.0339	0.0109	−0.0554	−0.0124	−30.995	0.0021
Direct effect (*c*′)	−0.0242	0.0122	−0.0482	−0.0001	−1.9791	0.0488
*a*_1_	−0.1036	0.0663	−0.234	0.0269	−15.631	0.1192
*a*_2_	0.3978	0.0644	0.2710	0.5246	6.177	0.000
*a*_3_	0.3324	0.0695	0.1956	0.4692	4.784	0.000
*b*_1_	0.0077	0.0118	−0.0155	0.0309	0.6537	0.513
*b*_2_	−0.0155	0.0108	−368	0.0059	−1.426	0.154
*b*_3_	−0.0088	0.0103	−0.0291	0.0116	−0.8509	0.3956
*d*_21_	0.5694	0.0591	0.4531	0.6858	9.636	0.000
*d*_31_	0.1417	0.0692	0.0054	0.2781	20.469	0.0417
*d*_32_	0.2923	0.0618	0.1707	0.4139	4.732	0.000
Indirect effects	Effects	HE	BootLLCI	BootULCI		
Total indirect effect	−0.0242	0.0050	−0.0199	−0.0002		
**Model 2 (Humiliation Victimization)**						
**Path**	**Coefficient**	**HE**	**BootLLCI**	**BootULCI**	**t**	***p***
Total effect (*c*)	−0.0281	0.0112	−0.0501	−0.0061	−2.5128	0.0126
Direct effect (*c*′)	−0.0193	0.0125	−0.0439	0.0053	−1.5439	0.1238
*a*_1_	−0.1036	0.0663	−0.234	0.0269	−1.5631	0.1192
*a*_2_	0.3978	0.0644	0.271	0.5246	6.1776	0.000
*a*_3_	0.3324	0.0695	0.1956	0.4692	4.7841	0.000
*b*_1_	0.0094	0.012	−0.0143	0.0331	0.778	0.4373
*b*_2_	−0.0208	0.0111	−0.0427	0.001	−1.8776	0.0615
*b*_3_	−0.0019	0.0106	−0.0227	0.0189	−0.1797	0.8576
*d*_21_	0.5694	0.0591	0.4531	0.6858	9.6368	0.000
*d*_31_	0.1417	0.0692	0.0054	0.2781	2.0469	0.0417
*d*_32_	0.2923	0.0618	0.1707	0.4139	4.7323	0.000
Indirect effects	Effects	HE	BootLLCI	BootULCI		
Total indirect effect	−0.0088	0.0055	−0.0198	0.0018		
**Model 3 (Detachment Victimization)**						
**Path**	**Coefficient**	**HE**	**BootLLCI**	**BootULCI**	**t**	***p***
Total effect (*c*)	−0.0473	0.0153	−0.0774	−0.0172	−3.0937	0.0022
Direct effect (*c*′)	−0.0226	0.0167	−1.3524	0.1774	−0.0556	0.0103
*a*_1_	−0.1036	0.0663	−0.2340	0.0269	−1.563	0.1192
*a*_2_	0.3978	0.0644	0.271	0.5246	6.1776	0.000
*a*_3_	0.3324	0.0695	0.1956	0.4692	4.7841	0.000
*b*_1_	0.0203	0.0161	−0.0114	0.0521	1.2601	0.2087
*b*_2_	−0.0465	0.0149	−0.0758	−0.0172	−3.124	0.002
*b*_3_	−0.0163	0.0142	−0.0442	0.0116	−1.1515	0.2506
*d*_21_	0.5694	0.0591	0.4531	0.6858	9.636	0.000
*d*_31_	0.1417	0.0692	0.0054	0.2781	2.0469	0.0417
*d*_32_	0.2923	0.0618	0.1707	0.4139	4.7323	0.000
Indirect effects	Effects	HE	BootLLCI	BootULCI		
Total indirect effect	−0.0247	0.0084	−0.0419	−0.0088		
Ind2: *a*_2_*b*_2_	−0.0185	0.0068	−0.033	−0.0064		

Notes: Abbreviations: BootLLCI: bootstrapping lower limit confidence interval; BootULCI: bootstrapping upper limit confidence interval; HE: standard error. Model: 6. Y: sexual victimization/humiliation victimization/detachment victimization. X: BIS. M1: emotional attention. M2: emotional clarity. M3: emotional repair. Covariates: age and sex. *N* = 272.

## Data Availability

The dataset is available in the repository.
